# The Effect of Intravascular Laser Irradiation of Blood on Serum Biomarkers and Clinical Outcome in Knee Osteoarthritis Patients: A Double-Blind Randomized Control Trial

**DOI:** 10.3390/ijms252413608

**Published:** 2024-12-19

**Authors:** Yu-Chi Su, Yu-Ping Shen, Chih-Ya Chang, Ke-Ting Pan, Shih-Ming Huang, Liang-Cheng Chen

**Affiliations:** 1 Department of Physical Medicine and Rehabilitation, Tri-Service General Hospital, School of Medicine, National Defense Medical Center, Taipei 114, Taiwan; vickysu0110@gmail.com (Y.-C.S.); shiopping@gmail.com (Y.-P.S.); gradesboy@gmail.com (C.-Y.C.); 2 Graduate Institute of Aerospace and Undersea Medicine, National Defense Medical Centre, Taipei 114, Taiwan; pankt930@gmail.com; 3 Biochemistry Department, National Defense Medical Center, Taipei 114, Taiwan; shihming7102@gmail.com

**Keywords:** intravascular laser irradiation of blood, knee osteoarthritis, molecular mechanism, immunomodulation, anti-inflammation

## Abstract

Knee osteoarthritis (OA) is a prevalent degenerative joint disease globally, causing pain, stiffness, and disability. Intravascular laser irradiation of blood (ILIB) has been used for chronic pain and musculoskeletal disease. However, evidence on the clinical benefits and serum biomarkers post-ILIB therapy in knee OA is insufficient. We designed a double-blind randomized controlled trial to evaluate the clinical and biological outcomes of ILIB therapy for knee OA. Seventeen patients with knee OA were randomly assigned to the ILIB and control groups. The outcomes included the Western Ontario and McMaster Universities Osteoarthritis (WOMAC) Scale, visual analog scale, and biomarker analysis of interleukin (IL)-6, IL-13, IL-1β, epidermal growth factor, macrophage inflammatory protein-1β, and eotaxin. The measurements were performed at baseline and three days, one month, and three months post-intervention. The ILIB group showed a significant improvement in the WOMAC-pain score at one month of follow-up than the control group. IL-1β levels reduced significantly on day three, one month, and three months, and IL-13 levels reduced on day three and three months during follow-up in the ILIB group. ILIB therapy reduced knee OA pain for one month and significantly reduced serum IL-1β and IL-13 levels, suggesting potential for pain management.

## 1. Introduction

Knee osteoarthritis (OA) is a common degenerative joint disease and is the primary cause of pain, stiffness, deformity, and disability among the elderly worldwide [1]. The incidence of knee OA is influenced by various risk factors, including older age, female sex, overweight or obesity, occupation, varus or valgus alignment, and sports injuries [2,3]. The causes of knee OA are multifactorial, primarily attributed to repetitive mechanical loading that triggers an inflammatory process within the knee joint, leading to the loss of chondrocytes, cartilage destruction, subchondral bone remodeling, and synovial inflammation [4,5]. Several inflammatory cytokines, including interleukin (IL)-1β, tumor necrosis factor (TNF)-α, IL-6, and IL-8, are involved in the process of cartilage degradation [6,7]. Many chemokines are overexpressed in OA, including CCL19, CCR7, MCP-1, and MIP-1, which have also been associated with varying degrees of knee pain [7,8].

The primary approach to knee OA involves non-surgical treatments, including weight loss, oral analgesic medications, physical therapy, intra-articular injection of corticosteroids, hyaluronic acid, or platelet-rich plasma [9,10]. Non-surgical treatments are effective in the early stage of knee OA (Kellgren and Lawrence grades I–III) [11]. However, oral analgesic drugs, such as acetaminophen, non-steroid anti-inflammatory drugs, or opioids, may demonstrate adverse effects. Furthermore, intra-articular injection of corticosteroids only exhibits short-term effects [9,12]. Regarding the therapeutic effects of platelet-rich plasma or hyaluronic acid, strong evidence confirming their benefits is lacking [13,14]. Typically, surgical intervention is required for the advanced stage of knee OA (Kellgren and Lawrence grade IV) or poor response to conservative treatment [9,10,15]. However, older patients with comorbidities face elevated surgical risks because of increased morbidity and mortality. Hence, investigators are continuously exploring effective non-surgical treatments for patients who do not respond to traditional conservative treatment or are contraindicated for surgery [16].

Laser irradiation of blood (ILIB) was first introduced by scientists in the former Soviet Union in 1981 [17]. In this method, a low-level laser directly irradiates blood via an optical fiber venous catheter [18]. ILIB has the potential to improve both the capacity and deformability of erythrocytes, leading to enhanced oxygen transport [17,19,20]. Furthermore, the photodynamic reactions decrease blood viscosity by inhibiting platelet aggregation and adhesion [19]. ILIB was initially applied in the treatment of cardiovascular diseases, aiming to enhance blood flow and optimize the function of blood cells [17]. ILIB can modulate the immune system by improving the mitochondrial function of white blood cells [21]. It also influences the cellular oxidation-reduction reactions in the respiratory chain by activating the mitochondria, leading to the modulation and production of immune proteins such as immunoglobulins, interferons, and interleukins [20]. One animal study also demonstrated that inflammatory cytokines, such as IL-1α, IL-1β, and IL-6, decrease significantly after low-level laser therapy [22]. To date, ILIB has been extensively applied to many diseases, such as stroke, fibromyalgia, insomnia, spinal cord injuries, myofascial pain, and multiple sclerosis [17,19,20,23]. A retrospective analysis revealed that ten sessions of ILIB treatment can significantly improve pain and sleep quality in patients with musculoskeletal disorders, including spondylopathies, enthesitis, tendinitis, periostitis, and osteoarthritis [23]. One case report also showed improvement in muscle power, decreasing the level of homocysteine in one 62-year-old patient diagnosed with subacute ischemic stroke [24]. However, evidence regarding clinical benefits and serum biomarkers of ILIB therapy in patients with knee OA is still lacking.

We hypothesize that ILIB therapy may decrease the inflammation, mediate the inflammatory cascade, and further decrease pain and disability in patients with knee OA. ILIB therapy may be a suitable choice for patients with knee OA, especially those who demonstrate a poor response to conservative treatment or are not suitable candidates for surgical intervention. Therefore, this study aimed to evaluate the clinical and biological effectiveness of ILIB therapy for knee OA.

## 2. Results

**Figure 1:**
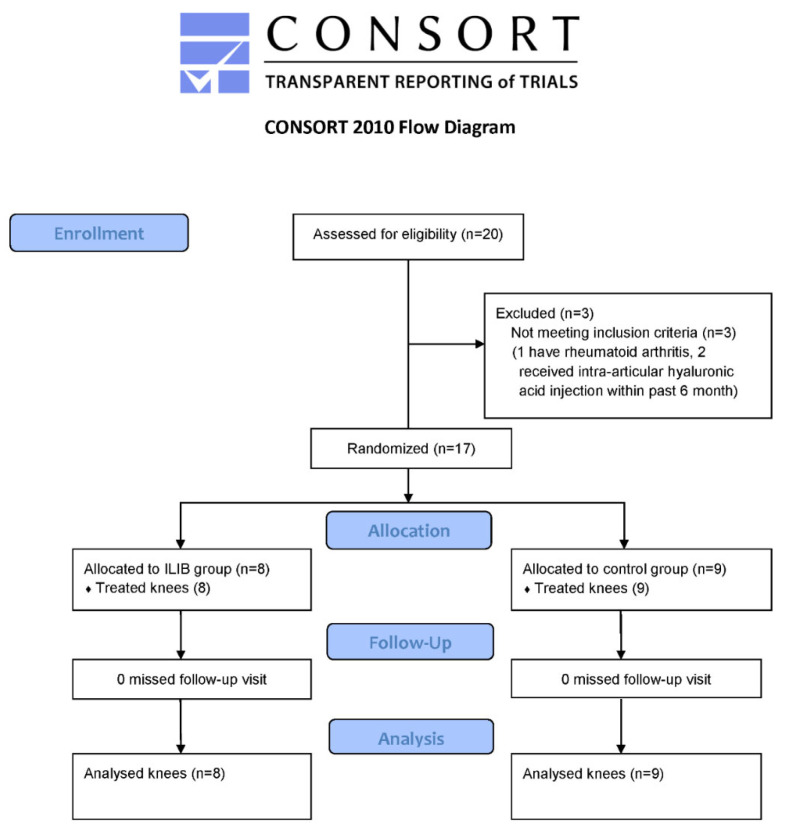
Study flow diagram.

A total of 20 participants diagnosed with single or bilateral knee OA were eligible for the study; of these, 17 were ultimately enrolled (Figure 1). These 17 participants were randomized into the ILIB (*n* = 8) and control (*n* = 9) groups (Figure 1). All participants completed the intervention and follow-up outcome evaluation. The missing data are omitted from the analysis. The mean age of the patients was 63 ± 7.0 and 61 ± 7.3 years in the ILIB and the control group, respectively (Table 1). No significant differences were noted in the baseline demographics and clinical conditions between the two groups, except for the baseline WOMAC function (Table 1).

As shown in Table 2, compared with baseline, WOMAC-pain and WOMAC-total scores at each follow-up time point revealed a significant reduction in both the ILIB and control groups (all *p* < 0.05). Furthermore, significant improvements in WOMAC-pain scores were observed in the ILIB group compared to the control group at the one-month follow-up (*p* = 0.027). At each follow-up time point compared to the baseline, Lequesne ’s index showed significant differences at one and three months in both groups. Compared to baseline, VAS scores at each follow-up time point revealed a significant difference in the control group, and the ILIB group showed a significant difference starting from the one-month follow-up assessment. However, the inter-group comparison of VAS and Lequesne ’s index at each time point between ILIB and the control group showed no significant difference (Table 3).

**Figure 2:**
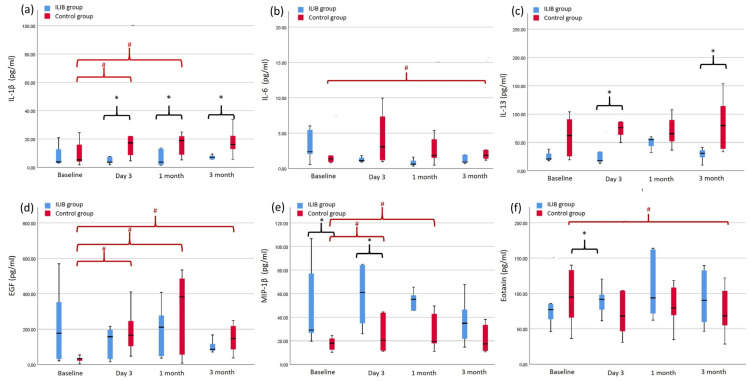
Biomarker assessment of ILIB and control group during follow-up time point: (**a**) IL-1β (**b**) IL-6 (**c**) IL-13 (**d**) EGF (**e**) MIP-1β (**f**) eotaxin; statistical analysis was performed using independent t-test or Mann–Whitney U test for intergroup comparisons (*: *p*-value < 0.05); Wilcoxon singled-rank test for intra-group comparisons to the baseline (#: *p* value < 0.05).

As shown in Figure 2, the concentrations of all biomarkers, except that of eotaxin, demonstrated a decrease at each follow-up time point compared to baseline in the ILIB group; however, the difference was not significant. In the control group, the concentrations of all biomarkers, except that of eotaxin, increased over time at each follow-up time point compared to baseline. The concentration of EGF increased significantly at all subsequent time points. The concentrations of IL-1β and MIP-1β increased significantly compared to the baseline after three days and one month. The concentrations of eotaxin and IL-6 showed significant differences compared to the baseline at the three-month follow-up. In contrast, the concentration of eotaxin increased over time in the ILIB group and decreased in the control group. For inter-group comparison, a significant difference was noted in IL-1β levels between the ILIB group and the control group on day three and after one and three months during follow-up (*p* = 0.018, 0.028, 0.043, respectively). A significant difference was noted in IL-13 levels in the ILIB group than those in the control group on day three and after three months during follow-up (*p* = 0.014, 0.012, respectively). Additionally, a significant difference in MIP-1β levels was noted between the two groups at baseline and three days after follow-up (*p* = 0.001, 0.021, respectively). No significant difference was noted in EGF, eotaxin, and IL-6 levels between the two groups. During the study period, none of the participants in this study had adverse events, and no additional conservative treatments were administered to any participant.

## 3. Discussion

To the best of our knowledge, the present study was the first double-blinded, randomized, controlled trial comparing the clinical efficacy and changes in serum cytokine levels associated with ILIB to those observed with sham laser in patients with knee OA. A statistically significant reduction in the VAS scores, Lequesne’s index, and WOMAC scores was noted in both the ILIB and control groups at each follow-up time point. However, only the WOMAC-pain subscales showed significant differences in the ILIB group than those in the control group at the one-month follow-up period.

In the biological analysis, we found that IL-1β levels showed significant reductions in the ILIB group than those in the control group on day three and after one and three months of follow-up. IL-1β is a well-known pro-inflammatory mediator in the pathogenesis of knee OA. It can activate the NF-κB pathway and further inhibit type II collagen expression, increase matrix metalloproteinases (MMP) production, suppress cartilage extracellular matrix synthesis, and promote cartilage catabolism [7,25]. IL-1β also stimulates the synthesis of inflammatory cytokines, such as IL-6 and TNF-α [7]. Furthermore, IL-1 was shown to induce apoptosis of chondrocytes [25]. Heraud et al. found that IL-1β could elevate the proportion of apoptotic cells in OA cartilage in a dose-dependent manner [26].

Two clinical trials have found that IL-1β inhibition in patients with painful knee and hip OA could have significant improvement in pain and function [25,27]. Therefore, the significant reductions in IL-1β levels for 3 months after ILIB therapy showed that this may lead to decreased apoptosis of chondrocytes, block synthesis of inflammatory cytokines, and further contribute to pain reduction and functional improvement.

IL-6 is a cytokine that exhibits both pro-inflammatory and anti-inflammatory functions, depending on different pathways [7,22]. IL-6 level is significantly elevated in the synovial fluid and serum samples in OA and is associated with cartilage loss [28,29,30,31]. Our study also revealed that IL-6 levels decreased over time in the ILIB group and increased in the control group; however, the differences were not significant in both groups. The aforementioned findings indicate that lower levels of IL-6 may slow the progression of knee OA and contribute to pain reduction. IL-13 levels decreased significantly in the ILIB group compared with those in the control group on day three and after three months during follow-up. IL-13 is a member of the T helper (Th) 2 family of cytokines, including IL-4, IL-5, IL-9, and IL-17 [32]. Tatsuya et al. demonstrated that IL-13 could stimulate periostin production by joint synoviocytes and further increase MMP levels, leading to cartilage destruction and knee OA progression [33]. Hence, lower IL-13 levels may positively impact knee OA progression and lead to clinical improvement.

Eotaxin and MIP-1β are members of the chemokine group. Both chemokines were shown to increase the receptors on chondrocytes, induce the expression of MMP, and play an important role in cartilage degradation [34,35]. Higher concentrations of MIP-1β and eotaxin have been reported in the synovial fluid of patients with OA [8,36]. Furthermore, patients with knee OA demonstrate higher plasma concentrations of eotaxin compared to healthy individuals [37]. Moreover, eotaxin levels in synovial fluid show a positive correlation to WOMAC severity [8]. MIP-1β levels exhibit a positive correlation with the severity of knee pain [38]. In this study, we found that the levels of MIP-1β decreased over time in the ILIB group and increased in the control group. A significant reduction in MIP-1β level in the ILIB group than that in the control group was noted on day three of the follow-up. This result suggested that lowering the MIP-1β level in the ILIB group may contribute to early pain reduction in patients with knee OA. On the other hand, we presume the level of eotaxin will decrease after ILIB therapy. Un-expectedly, our results revealed that eotaxin levels increased in the ILIB group at every follow-up time point, contrary to the control group, which demonstrated a reduction. A significant difference was found upon comparing the ILIB group to the control group at the three-month follow-up. Hence, elevated eotaxin levels in the ILIB group may offset the positive effects of ILIB in decreasing inflammatory cytokines. The exact mechanism of increasing eotaxin level after ILIB therapy remains un-known because of limited studies and data. Further research is needed to explore the association between eotaxin and ILIB therapy.

In our study, we also noticed a decrease in the concentration of EGF in the ILIB group, contrary to the increase in EGF levels in the control group at all follow-up time points. EGF can activate signal transduction via EGF receptors (EGFR) and further influence cell differentiation, proliferation, and migration [39]. Blocking the EGFR/mitogen-activated protein kinase (MAPK) cascade could reduce the TNF-α and IL-1β production in microglia in spinal cord injury [40]. N. Kazemikhoo1 et al. also reported that ILIB therapy could inhibit EGFR expression, which may reduce inflammation in diabetes patients [18]. Although no significant difference was noted in the EGF levels between the ILIB and control groups in patients with knee OA in our study, the decreased EGF expression may downregulate the EGFR/MAPK pathway, which further reduces inflammatory cytokine production and mitigates cartilage destruction in knee OA in the ILIB group.

Overall, our study demonstrated that ILIB therapy could significantly decrease IL-1β and IL-13 levels in knee OA patients. This result suggests that ILIB therapy has the potential to serve as an adjuvant treatment for other inflammatory diseases. Although the evaluated biochemical factors demonstrated positive effects on decreasing inflammation, only pain relief was significant in our study. Several factors may contribute to this outcome. First, multiple factors are involved in the progression of knee OA [41]. One randomized control study demonstrated that blocking the expression of IL-1β did not yield desired therapeutic effects in knee OA [41]. Second, both groups were subjected to intravenous catheter placement, potentially inducing a placebo effect in the control group. Hence, the actual clinical effect of ILIB therapy may be underestimated. There are some limitations to this study. First, the sample size was small; therefore, studies with larger participants are required in the future to confirm the therapeutic and biological effects of ILIB. Second, we only monitored the outcomes for three months. Hence, long-term follow-up may be also needed to evaluate the effects of ILIB therapy beyond three months. Third, we used a questionnaire for clinical outcome measurement, which was subjective. Objective knee functional tests, such as gait analysis, balance tests, or the timed up and go test, are needed in future studies. Finally, we administered only one course of ILIB therapy; one retrospective study analyzed pain scores and sleep quality in patients diagnosed with musculoskeletal disease who received three courses of ILIB therapy [23]. The results showed significant pain improvement and also suggested that ILIB may have an accumulative effect on pain control [23]. However, one RCT showed ILIB therapy could alleviate oxidative stress and mitochondrial dysfunction in chronic spinal cord injury patients, who received one course (1 h per day for 15 days) of ILIB therapy [21]. The most effective course of ILIB therapy remains controversial. Hence, further studies are needed to assess the accumulated therapeutic effect with multiple courses of ILIB therapy.

## 4. Materials and Methods

### 4.1. Study Design

This was a double-blind randomized controlled trial conducted at one medical center in Taiwan from 28 October 2020 to 16 February 2023. The study protocols were approved by the institutional review board of Tri-Service General Hospital (Taipei, Taiwan) and are listed at www.ClinicalTrials.gov (accessed on 20 October 2020), registration number: NCT04598854). Each participant agreed to and signed informed consent before involvement in this trial.

### 4.2. Participants

All participants were recruited from the outpatient clinic of the Department of Physical Medicine and Rehabilitation. One single physician collected clinical histories of the patients and performed all physical examinations. The inclusion criteria of the participant were as follows: (1) age between 50 and 75 years, (2) clear consciousness and ability to communicate, (3) a clinical diagnosis of knee OA with Ahlbäck classification grade I–III and a Visual Analog Scale (VAS) score of more than 4, (4) symptoms lasting for at least six months. The exclusion criteria were as follows: (1) previous intra-articular knee joint injection of hyaluronic acid or steroids within the past six months, (2) oral non-steroidal anti-inflammatory drugs or steroid treatment within the preceding week, (3) medical history of coagulopathy, rheumatoid arthritis, benign or malignant tumor, and metastatic tumor near the knee joint, and (4) surgical history of total knee replacement or lower extremity amputation. Participants were excluded if they met any of the exclusion criteria.

### 4.3. Intervention

Participants were randomly separated into the ILIB or control groups using the randomized number created on the computer with a 1:1 ratio. The ILIB group received intravenous helium-neon laser phototherapy at 632.8 nm (Classconn helium-neon Laser System, Medipark, Seoul, Republic of Korea). A vein-indwelling needle was placed in their veins located in the upper elbow, and the laser fiber catheter was introduced through the indwelling cannula. The power was set between 2.5 and 3.0 Mw for 60 min for each session, once a day for five consecutive days. The intervention steps of the control group were the same, except that the output power was adjusted to zero intensity. During the study, all participants were blinded to the group allocation. The participants did not receive any physical therapy, intra-articular injection, or oral analgesics during the study period, except for acetaminophen (500 mg, up to 4 g/day) as medication for pain control. An investigator regularly checked whether other types of therapies were utilized by patients at each follow-up time point during the study. No side effect of the intervention was noted during the study.

### 4.4. Outcome Measurements

All outcomes were evaluated before ILIB intervention, on day three, one month, and three months post-treatment by a single researcher who was blinded to the randomized allocation. The clinical outcomes included inter-group differences in the improvement of measurements prior to ILIB treatment and at three months post-therapy. For biological outcomes, 20 mL of autologous peripheral venous blood samples were drawn at each time point for blood tests. The blood was centrifuged at 4000 rpm for 10 min. Subsequently, the serum was separated and stored at −80 °C before analyzing the inflammatory biomarkers.

### 4.5. Primary Outcomes

#### Western Ontario and McMaster Universities Osteoarthritis (WOMAC) Index

WOMAC was developed by Bellamy in 1988 to evaluate the OA of the knees and hips according to the symptoms [42]. This index is evaluated via a self-administered questionnaire with a total of 24 items divided into three domains: pain (5 items), stiffness (2 items), and functioning (17 items) [42,43]. Each item is scored on a scale of 0–4 (0 = no difficulty, pain, or stiffness; 4 points = worst pain and stiffness) [43]. WOMAC has demonstrated good reliability and validity in evaluating the severity and treatment outcomes of knee and hip OA [44].

### 4.6. Secondary Outcomes

#### VAS

The VAS was utilized to measure pain intensity with scores ranging from 10 (worst pain) to 0 (no pain) [45]. The minimum clinically important difference for pain severity was defined as a decrease in at least 2 of the VAS scores [46].

### 4.7. Lequesne’s Severity Index

The Lequesne’s severity index was developed as an interview format to evaluate the OA in the 1980s [47]. This index includes 11 questions about pain, discomfort, and function [30]. The score ranges from 0 (no pain, no disability) to 24 (maximum pain and disability) [47].

### 4.8. Inflammatory Biomarkers Assessment

Blood samples were collected from each participant following a standardized protocol according to clinical practice. Briefly, serum samples were separated from the whole blood by centrifugation at 4000 rpm. Subsequently, the levels of IL-6, IL-13, IL-1β, epidermal growth factor (EGF), macrophage inflammatory protein (MIP)-1β, and eotaxin were measured in the serum samples using the sandwich ELISA method according to the manufacturer’s instructions (eBioscience, San Diego, CA, USA).

### 4.9. Data Analyses

SPSS statistics version 22 (IBM, Armonk, NY, USA) was used to analyze all collected data. Continuous and categorical demographic data were analyzed using independent t-test, Mann–Whitney *U*, chi-squared, or Fisher’s exact tests. The independent t-test or Mann–Whitney *U* test was used for intergroup comparisons of mean differences across time points, whereas the Wilcoxon singled-rank test was used for intragroup comparisons to the baseline. The statistical significance was set at a *p*-value < 0.05.

## 5. Conclusions

The study demonstrated that ILIB therapy could decrease IL-1β and IL-13 concentration in blood circulation and may relieve pain in patients with knee OA. Further studies with larger sample sizes are required to verify its therapeutic efficacy.

## Figures and Tables

**Table 1:** Demographic information.

	ILIB Group (*n* = 8)	Control Group (*n* = 9)	^a^ *p* Value
Gender, *n* (%)			0.620
Female	6 (75)	5 (55.6)	
Male	2 (25)	4 (44)	
Age (year) ± SD (range)	63.13 ± 7.0 (53–71)	61.56 ± 7.3 (54–74)	0.066
BH (cm) ± SD (range)	158.8 ± 9.5 (153–176)	164.5 ± 9.4 (150–176)	0.238
BW (kg) ± SD (range)	61.5 ± 11 (48–75)	67.3 ± 16 (50–74)	0.412
DM (%)	1 (12.5)	1 (11.1)	1.000
Hypertension (%)	3 (37.5)	3 (33.3)	1.000
VAS ± SD	4.1 ± 3.0	5.0 ± 2.9	0.541
WOMAC pain ± SD	15.63 ± 5.2	13.78 ± 7.2	0.562
WOMAC stiffness ± SD	4.13 ± 3.18	5.56 ± 3.71	0.410
WOMAC function ± SD	20.8 ± 8.4	39.2 ± 17.48	0.017
WOMAC total ± SD	40.5 ± 10.7	58.5 ± 26.3	0.091
Lequesne’ Index ± SD	7.63 ± 2.7	8.11 ± 4.5	0.799
EGF (pg/mL) ± SD	213.9 ± 200.4	49.9 ± 66.7	0.093
Eotaxin (pg/mL) ± SD	80.2 ± 28.9	95.6 ± 39.2	0.378
IL-1β (pg/mL) ± SD	7.91 ± 8.7	9.9 ± 8.1	0.696
IL-6 (pg/mL) ± SD	3.28 ± 2.35	2.22 ± 2.42	0.429
IL-13 (pg/mL) ± SD	42.36 ± 52.04	141.8 ± 247.6.	0.057
MIP-1β (pg/mL) ± SD	49.04 ± 32.93	17.22 ± 5.46	0.001

SD = standard deviation; BH = body height; BW = body weight; DM = diabetes mellitus; VAS = visual analogue scale; WOMAC = the Western Ontario and McMaster Universities Arthritis Index; EGF = epidermal growth factor; MIP = macrophage inflammatory protein; IL = interleukin; ^a^ Mann–Whitney *U* test, chi-squared test, or Fisher’s exact test.

**Table 2:** Comparison of changes in WOMAC total and subscales between both groups.

	ILIB Group (*n* = 8) Mean ± SD Mean Difference ± SD		^a^ *p* Value	Control Group (*n* = 9) Mean ± SD Mean Difference ± SD		^a^ *p* Value	^b^ *p* Value
**WOMAC (pain) baseline**	**15.63 ± 5.263**			13.78 ± 7.2			
Day 3	7.25 ± 3.308	−8.37 ± 6.11	0.011	6.0 ± 5.148	−7.77 ± 3.6	0.008	0.888
Month 1	2.50 ± 2.204	−13.12 ± 5.93	0.012	6.33 ± 5.5	−7.44 ± 2.6	0.007	0.027
Month 3	5.5 ± 5.155	−10.12 ± 7.29	0.012	5.0 ± 5.127	−8.62 ± 3.42	0.012	0.878
WOMAC (stiffness) baseline	4.13 ± 3.182			5.56 ± 3.71			
Day 3	2.5 ± 2.268	−1.625 ± 2.38	0.047	4.44 ± 4.157	−1.11 ± 2.57	0.205	0.963
Month 1	1.88 ± 2.357	−2.25 ± 3.61	0.127	4.44 ± 3.321	−1.11 ± 1.8	0.112	0.743
Month 3	1.63 ± 2.2	−2.50 ± 2.39	0.017	3.5 ± 4.84	−1.50 ± 2.97	0.147	0.574
WOMAC (function) baseline	20.88 ± 8.442			39.2 ± 17.48			
Day 3	15.25 ± 10.22	−5.62 ± 7.92	0.093	25.56 ± 20.2	−13.6 ± 13.9	0.028	0.093
Month 1	6.0 ± 7.72	−14.8 ± 9.58	0.012	27.67 ± 20.8	−11.5 ± 14.6	0.005	0.888
Month 3	11.0 ± 9.53	−9.87 ± 10.6	0.005	21.38 ± 23.6	−17.1 ± 19.0	0.005	0.279
WOMAC (Total) baseline	40.5 ± 10.7			58.56 ± 26.33			
Day 3	25.0 ± 11.4	−15.5 ± 11.21	0.012	36 ± 26.05	−22.55 ± 16.08	0.015	0.236
Month 1	10.75 ± 9.96	−29.7 ± 14.88	0.012	38.44 ± 28.47	−20.11 ± 15.78	0.018	0.321
Month 3	18.13 ± 14.6	−22.37 ± 16.73	0.012	29.88 ± 32.1	−27.25 ± 13.18	0.025	0.442

SD = standard deviation, WOMAC = the Western Ontario and McMaster Universities Arthritis Index; ^a^ Friedman test with Wilcoxon signed-rank post hoc analysis (each time point versus baseline); ^b^ Mann–Whitney *U* Test (mean difference, intergroup).

**Table 3:** Comparison of changes in VAS and Lequesne ’s index between both groups.

	ILIB Group (*n* = 8) Mean ± SD Mean Difference ± SD		^a^ *p* Value	Control Group (*n* = 9) Mean ± SD Mean Difference ± SD		^a^ *p* Value	^b^ *p* Value
**VAS baseline**	**4.12 ± 3.07**			5.0 ± 2.98			
Day 3	2.56 ± 2.63	−1.81 ± 1.86	0.075	3.27 ± 2.53	−1.72 ± 1.8	0.027	0.963
Month 1	1.31 ± 1.83	−2.81 ± 2.46	0.018	3.61 ± 2.73	−1.38 ± 1.53	0.043	0.236
Month 3	1.62 ± 2.44	−2.5 ± 2.12	0.017	2.93 ± 2.48	−2.68 ± 1.86	0.024	0.574
Lequesne ’s index baseline	7.63 ± 2.77			8.11 ± 4.59			
Day 3	5.00 ± 2.77	−2.62 ± 3.2	0.051	5.22 ± 4.91	−2.88 ± 3.62	0.058	0.815
Month 1	2.88 ± 2.23	−4.75 ± 3.15	0.018	5.00 ± 4.52	−3.11 ± 3.1	0.028	0.370
Month 3	3.25 ± 2.60	−4.37 ± 3.46	0.018	4.13 ± 3.48	−3.62 ± 2.92	0.021	0.798

SD = standard deviation, VAS = visual analog scale; ^a^ the Wilcoxon signed-rank post hoc analysis (each time point versus baseline); ^b^ Mann–Whitney *U* test (mean difference, inter-group).

## Data Availability

Data is contained within the article.
